# Escherichia coli Meningitis in a Patient With Urinary Tract Infection: A Case Report

**DOI:** 10.7759/cureus.41312

**Published:** 2023-07-03

**Authors:** Aglisa Memia, Xheni Deda, Andrea Broka, Mariana Kawalet, Judith Berger

**Affiliations:** 1 Medicine, St. Barnabas Hospital (SBH) Health System, Bronx, USA; 2 Hepatology, Missouri University Hospital, Columbia, USA; 3 Nephrology, University of California, Davis, Davis, USA; 4 Infectious Diseases, St. Barnabas Hospital (SBH) Health System, Bronx, USA

**Keywords:** escherichia coli, urinary tract infection, diabetic ketoacidosis, alcohol use disorder, dka, uti, gram negative bacteremia, pyelonephritis, meningitis, e. coli

## Abstract

This article discusses a case of *Escherichia coli* (E. coli) meningitis resulting in altered mental status in a patient with multiple pre-existing comorbidities. The case highlights the underestimated risk of community-acquired gram-negative meningitis in adults, which can have a high mortality rate, particularly in elderly patients with sepsis and urinary tract infections. Diagnosis of *E. coli* meningitis was confirmed by analyzing cerebrospinal fluid obtained through the lumbar puncture and blood cultures. Treatment involved prompt administration of antibiotics and supportive care. However, the emergence of antibiotic resistance, such as extended-spectrum beta-lactamase production, in community-acquired *E. coli* meningitis is an increasing concern. Therefore, early recognition and appropriate management are crucial in the diagnosis and treatment of this life-threatening condition.

## Introduction

Acute bacterial meningitis is a severe and potentially fatal infection that affects the leptomeninges. Gram-negative bacilli, including *Escherichia coli* and *Klebsiella pneumonia*, can rarely cause community-acquired bacterial meningitis, with a prevalence ranging from 3% to 9% [1-4]. Prompt recognition and appropriate management are crucial in the diagnosis and treatment of this life-threatening condition, which typically presents with fever, headache, neck stiffness, altered mental status, and seizures.

Diagnosis is confirmed through cerebrospinal fluid (CSF) analysis obtained via lumbar puncture (LP), and treatment involves the prompt administration of antibiotics and supportive care. The management of bacterial meningitis can be complex, particularly in elderly patients with comorbidities [4,5]. In this article, we present a case of *E. coli* meningitis leading to altered mental status in a patient with a urinary tract infection (UTI), highlighting the importance of vigilance in the diagnosis and management of this potentially fatal infection.

## Case presentation

A 63-year-old man with a history of hypertension, hyperlipidemia, type 2 diabetes mellitus, and alcohol use was brought to the emergency room with acute mental status changes reported by his wife. He had a heart rate of 126 beats/minute, blood pressure of 140/89 mmHg, and a respiratory rate of 24/minute. He was afebrile, but his capillary blood glucose (CBG) was high. He had dry mucous, and the heart/lung/abdomen exam was unremarkable. His Glasgow Coma Scale (GCS) score was 10 (eye opening = 3, verbal response = 2, and motor response = 5), with no signs of meningeal irritation or focal neurological deficits. Laboratory tests showed left leukocytosis (WBC = 12,000/uL), thrombocytopenia (platelets = 60,000/uL), metabolic acidosis (bicarbonate = 12 mEq/L) with anion gap at 22 mEq/L, serum glucose at 1120 mg/dL, blood urea nitrogen (BUN)/creatinine at 61/3.7 mg/dL, alanine aminotransferase (ALT) at 52 IU/L, aspartate aminotransferase (AST) at 81 IU/L, total bilirubin at 2.5 mg/dL, alkaline phosphatase (ALP) at 97 IU/L, moderate serum ketones, and a blood osmolality of 331 mOsm/L.

**Figure 1:**
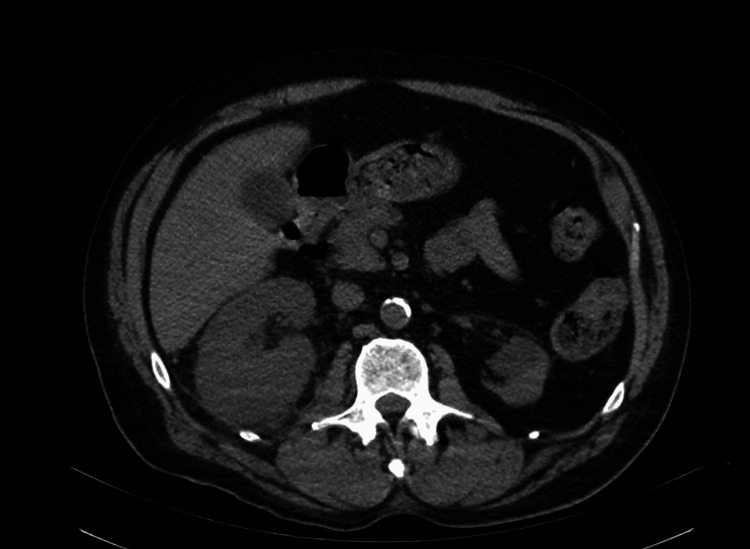
CT of the abdomen/pelvis with contrast showed evidence of acute right pyelonephritis

The patient was admitted with a diagnosis of acute encephalopathy due to diabetic ketoacidosis (DKA) and sepsis secondary to *E. coli* bacteremia resulting from a UTI. He was initially treated with broad-spectrum antibiotics, cefepime, and metronidazole, due to evidence of sepsis. Once blood culture sensitivity results were available, antibiotics were narrowed to ceftriaxone. On day four of antibiotic therapy and correction of DKA, there was no improvement in mental status, and an LP was performed. The results confirmed meningitis, with laboratory findings in the CSF, as shown in Table 1. An MRI of the brain ruled out the presence of a brain abscess, while a CT of the abdomen/pelvis with contrast showed evidence of acute right pyelonephritis (Figure 1). Ciprofloxacin was added to the antibiotic regimen for better urinary tract penetration. After five days of treatment with ceftriaxone and a repeat blood and urine culture, there was no bacterial growth. A repeat LP at two weeks of antibiotics (Table 2) resulted in CSF that was negative for WBC and continued to be negative for bacterial growth. The patient completed a total of 21 days of ceftriaxone plus a course of 14 days (about two weeks) of ciprofloxacin for a complicated UTI. The patient achieved full recovery from meningitis with a return of full functionality as prior to presentation.

**Table 1:** CSF laboratory results before starting antibiotics

WBC	5954/mm3
Neutrophils	80%
Lymphocytes	19%
RBC	15/mm3
Protein	50 mg/dL
Glucose	83 mg/dL

**Table 2:** CSF laboratory results after two weeks of antibiotic therapy

WBC	1/mm3
RBC	1/mm3
Protein	27 mg/dL
Glucose	83 mg/dL

## Discussion

Spontaneous community-acquired gram-negative meningitis is an under-recognized cause of meningitis in adults that carries significant mortality, particularly in elderly patients presenting with UTI and sepsis [6]. *E. coli* is one of the most reported organisms in community-acquired bacterial meningitis [7]. Various risk factors like trauma, neurosurgical procedures, alcoholism with liver cirrhosis, uncontrolled diabetes, disseminated strongyloidiasis, HIV, and chronic organ dysfunction have been reported as a cause of *E. coli* meningitis [8,9]. Community-acquired *E. coli* meningitis has been identified with documented primary *E. coli* infection from the urinary tract and gastrointestinal tract, and a rare case report has identified the source from a retropharyngeal abscess [10].

In the case we encountered, the source was concluded to be pyelonephritis complicated with bacteremia in a patient with uncontrolled DM and a history of alcohol use disorder. Although *E. coli* in the CSF was not identified, likely explained by antibiotic administration prior to CSF collection, our patient had enough evidence of acute bacterial meningitis based on the results of CSF studies and positive blood cultures.

It has been noted that wild-type sensitive *E. coli* causes meningitis in up to 20% of cases [5,8]. The emergence of antibiotic resistance is a rising concern. Extended-spectrum beta-lactamase (ESBL) production was present in up to 7% of cases of community-acquired *E. coli* meningitis and up to 9% of cases showed broad-spectrum beta-lactamase production [10]. In our patient, the organism was a broad-spectrum beta-lactamase producer, sensitive to third-generation cephalosporins. A three to four-week duration of treatment was considered adequate for meningitis without brain abscess.

Mortality with community-acquired *E. coli* meningitis remains high and ranges from 50% to 90%, reaching 85% in disseminated strongyloidiasis and up to 100% in the case of liver cirrhosis [8,11]. The presence of multi-organ dysfunction worsens the chances of recovery [11]. A high index of suspicion and considering LP, when initial treatment fails, can prevent morbidity and mortality.

## Conclusions

In conclusion, acute encephalopathy in the elderly can be a challenging diagnosis and given the high prevalence of urinary tract infections, complicated by sepsis, with co-existing comorbidities such as diabetes mellitus, the presence of meningitis can be missed and, therefore, an LP will be warranted for the diagnosis of meningitis. Prompt diagnosis and appropriate antibiotic therapy are crucial to improving the chances of recovery. With the emergence of antibiotic resistance, it is essential to identify the organism's susceptibility to appropriate antibiotics. Extended duration of treatment may be necessary in some cases, particularly for those with brain abscesses.
